# Opportunities and Challenges to Linkage to Housing in the Context of a Sexual and Reproductive Health Program for Youth Experiencing Homelessness

**DOI:** 10.1007/s11121-023-01560-y

**Published:** 2023-06-10

**Authors:** Maisha R. Huq, Danielle R. Phillips, Christine Childers, Rebecca Chavez, Jacqueline Tellei, Lenora Blakely, Elizabeth M. Aparicio

**Affiliations:** 1 Department of Behavioral and Community Health, School of Public Health, University of Maryland, College Park, 4200 Valley Drive, 20742 College Park, MD USA; 2 School of Social Work, University of Maryland, Baltimore, MD USA; 3 Waikiki Health, DOD Department of Public Health, Fayetteville, NC USA; 4 Waikiki Health, Honolulu, HI USA; 5 PATH Clinic, Waikiki Health, Honolulu, HI USA; 6 Community THRIVES Lab, Department of Behavioral and Community Health, School of Public Health, University of Maryland, College Park, MD USA

**Keywords:** Youth homelessness, Housing, Sexual and reproductive health, Primary prevention

## Abstract

Youth homelessness remains a major public health issue in the USA, and youth experiencing homelessness (YEH) are still one of the more understudied and underserved groups. Comprehensive sexual and reproductive health (SRH) programs for YEH are rare. Yet, such programs are potentially effective settings through which to link YEH with housing services. Wahine (“woman”) Talk is one such comprehensive program for YEH, and is a multilevel intervention delivered out of a youth drop-in center in Honolulu, Hawai ‘i. One of Wahine Talk’s core components is addressing basic needs, including providing linkages to housing services. Little research exists on SRH programs’ opportunities and challenges to providing linkage to housing for YEH. The current study is an exploratory study asking, “What are opportunities and challenges to linking young women experiencing homelessness to housing services through a comprehensive sexual and reproductive health program?” The study team collected in-depth qualitative data through seven focus groups and 25 individual interviews with Wahine Talk staff and youth participants aged 14–22 years. Multiple team members analyzed the data using template analysis. The analysis revealed that while comprehensive SRH programs may have some opportunities and challenges to linking YEH to housing services that are consistent with traditional housing assistance programs, there are also factors specific to SRH programs. In particular, opportunities would be SRH programs employing a housing staff person and bolstering staff-youth meetings and communication. A challenge specific to SRH programs would be prioritizing youth’s reproductive justice (i.e., choice) in lieu of solely prioritizing pregnancy reductions and delays; thus, it is recommended to train staff to prioritize youth’s reproductive justice. The findings highlight the importance of SRH programs having staff focused on housing, sufficient opportunities for youth and staff to communicate with each other, and staff trained to prioritize youth’s reproductive justice.

## Introduction

Homelessness remains a major public health issue in the USA, with youth experiencing homelessness (YEH) still one of the more understudied and underserved groups (Morton et al., 2018). Each year, approximately 3.5 million unaccompanied youth between 18 and 25 years of age experience homelessness (Morton et al., 2018). This translates to one in 10 individuals in this age group experiencing homelessness (Morton et al., 2018).

Traditional avenues to link YEH to housing or housing services in the USA include youth entering a homeless shelter (Slesnick et al., 2016). Homeless shelters are often the first point of service contact for individuals experiencing homelessness (Slesnick et al., 2016). Depending on the time of year, the shelter often provides an overnight bed, meals, and referrals to more stable housing services or other wraparound services. However, only 20–30% of YEH report ever having stayed at a shelter (Slesnick et al., 2016). YEH may avoid visiting a shelter for a multitude of reasons, including adversarial interactions with other individuals experiencing homelessness and because the shelter is not tailored to the youths’ unique developmental needs (Ha et al., 2015).

Another common avenue for connecting YEH to housing or housing services is through drop-in centers providing housing services (Pederson et al., 2016). Drop-in centers refer to centers which provide YEH with basic needs (e.g., showers, meals, clothing). There are also drop-in centers which provide higher-level wrap-around services such as linkage to housing, mental health and substance use treatment referrals or services, and other social and health supports. Relative to homeless shelters which tend to have more restrictive rules, drop-in centers embody a “come as you are” approach, and due to this, are quite popular (Pederson et al., 2016).

The school setting is a setting which has successfully provided linkage to housing services for YEH despite lacking a primary focus on housing (Le Viramontes et al., 2019). The McKinney Act’s Education of Homeless Children and Youth (EHCY) program requires all school districts to support YEH (Cunningham et al., 2010) through Homeless Liaisons, who are specific district-level K-12 staff who work to assist YEH with their access to housing, transportation, and other education-related issues (Le Viramontes, 2019). Every federally funded school district employs at least one homeless liaison. The liaison may have other roles and duties, yet works with other district administrators, school administrators, the transportation department, and food services staff (Le Viramontes, 2019). Research has found that such liaisons often serve as “natural mentors” for the youth and are facilitators for connecting youth with housing and other services in a school context (Le Viramontes, 2019).

Similar to schools, comprehensive sexual and reproductive health (SRH) programs lack a primary focus on housing. Comprehensive SRH programs’ primary mission is to provide SRH services; however, they also provide multiple wrap-around services. In contrast, standalone SRH programs provide only SRH services. Although comprehensive SRH programs are rare, there is a need to utilize comprehensive SRH programs with this population because YEH, in addition to being homeless, bear a disproportionate burden of all youths’ SRH risks. Housing can reduce the prevalence of SRH risk (Dickson-Gomez et al., 2017; Kumar et al., 2015; Wenzel et al., 2007; McNeill et al., 2020; Leifheit et al., 2019). Thus, through providing linkage to housing services, comprehensive SRH programs provide an opportunity to reduce both housing insecurity and SRH risk in this population. YEH are more likely than their housed peers to engage in risky sexual behaviors, such as unprotected and survival sex (Alessi et al., 2021; Greene et al., 1999; Heerde et al., 2014), where survival sex refers to need-driven sex in exchange for goods, including housing. Female YEH, including runaway youth, have a four times greater likelihood of becoming pregnant than their non-runaway peers (Berry et al., 2000; Dworsky et al., 2018). Lesbian, gay, bisexual, transgender, and queer youth are heavily overrepresented among the YEH population, and lesbian, gay, bisexual, transgender, and queer YEH are at significantly greater risk of experiencing exploitive survival sex compared to heterosexual and cisgender YEH peers (Walls & Bell, 2011).

There is limited research on how to connect YEH to housing services through comprehensive SRH programs despite housing security and SRH being intertwined. Although comprehensive SRH programs for YEH are rare, have only a secondary focus on housing, and have only a nascent research base (Aparicio et al., 2018, 2019a, 2019b, 2021), they are potentially an effective way to link YEH with housing services. Further study is needed on opportunities and challenges to linkage to housing services in this setting.

The current work is an exploratory study utilizing qualitative data to better understand opportunities and challenges to accessing housing services for YEH in the context of an innovative comprehensive SRH program (Wahine “woman” Talk). The research question is, “What are opportunities and challenges to linking young women experiencing homelessness to housing services through a sexual and reproductive health program?”.

## Method

### The Intervention

Wahine Talk is a multi-level sexual reproductive health program operating at the individual, interpersonal, and sexual health care system levels. Its primary aim is to link YEH to sexual health care, improving their birth control education, access, and use. Given its comprehensive nature, however, its four core components are (1) basic needs and social services, (2) peer mentorship, (3) sexual health education groups, and (4) linkage to and provision of sexual healthcare. Thus, Wahine Talk provides drop-in center open hours, basic needs, and wrap-around services such as linkage to housing and employment services in addition to its sexual healthcare services. Set at a youth drop-in center (Youth Outreach; YO!) institutionally linked to a federally qualified health care center (Waikiki Health); Wahine Talk was delivered by an interdisciplinary team.

Individuals were eligible for intervention enrollment if they were experiencing homelessness or at imminent risk for homelessness (e.g., insecurely housed or on frequent runaway status), biologically female (including youth of any gender identity or gender expression who had a uterus), and aged 14–22 years. Individuals were not eligible if they were currently pregnant or using long-acting reversible contraceptive (LARC) at enrollment. The eligibility criteria were selected to recruit female youth experiencing homelessness as homeless youth are a high-risk group for adolescent pregnancy. Individuals 14–22 years old being served by our community partner were legally allowed to consent to their own SRH services in Hawai ‘i. Thus, 14–22 was selected as the age eligibility for the intervention.

All intervention participants enrolled via a modified informed consent process wherein participants were allowed to consent themselves if at least 14 years old (i.e., even if under 18) due to a legal ability to consent to all SRH education and services being offered in the program and, frequently, due to estrangement from their parent/guardian. Once enrolled, youth participants completed a pre-intervention quantitative interview. Then, over the course of 23 weeks, participants completed weekly sexual health education group sessions at YO! on various SRH topics. The sessions were 1–1.5 h in duration and varied in size, ranging 0 to 14 participants, and were led by 1–2 health educators.

Outside of the group sexual health education sessions, youth participants interacted with Wahine Talk staff through a Wahine Talk–given cellphone, drop-in hours, and clinic visits. Specifically, at enrollment, all participants received a basic smartphone and data boosts on a tiered system to facilitate program engagement and communication with their Peer Mentor, a full-time Wahine Talk staff who led sexual health education group sessions and was employed to be available at all times by phone for the youth participants. The individual was referred to as a peer mentor because they were similar in age to the participants and functioned as a mentor. Participants engaged with their Wahine Talk peer mentor and other participants through text, social media direct messages, and social media private group posts. A total of 3781 texts were exchanged between Wahine Talk staff and participants, where the average participant texts with the peer mentor was 26.4, 45.4, and 56.7 texts for cohorts 1, 2, and 3, respectively.

Drop-in hours for basic needs services such as a shower, locker, meals, laundry, and a safe space were available an average of 12 h per week. Youth participants were also provided with bus passes and “pick-ups” to attend the program. All services were offered on a continuing basis after enrollment. Additional details about the intervention are published elsewhere (). The Institutional Review Board at the University of Hawai ‘i approved this study.

### Sample

The study eligibility criteria were the same as the intervention eligibility criteria. All Wahine Talk intervention providers (synonymously referred to as “staff”) and youth participants were invited to the study’s qualitative data collection. In specific, there were three cohorts of Wahine Talk. For each cohort, the consent forms at enrollment described the qualitative data collection (e.g., purpose, risks, benefits, compensation). Thus, all Wahine Talk youth participants consented to the qualitative data collection at enrollment. Then, at intervention completion, the cohort’s Wahine Talk staff and youth were invited to the focus groups and interviews. Each participant received $50 as compensation for completing the focus group or interview.

All Wahine Talk staff participated in the qualitative data collection (*n* = 7). One provider focus groups was conducted for each of the three Wahine Talk cohorts, totaling three provider focus groups. Thus, while the Wahine Talk staff changed throughout the intervention period, all Wahine Talk staff present at the time of data collection participated in the focus groups. Each focus group included *n* = 4 providers, all female, typically a health educator, peer mentor, program manager, and medical provider.

Of all 68 intervention youth participants, 35 of the 68 participated in the qualitative data collection for this study (six of whom participated in both a focus group and individual interview). We confirmed there are no significant differences in the demographic characteristics nor study outcomes (i.e., including linkage to housing) between the intervention and study samples. Table 1 provides sample characteristics of Wahine Talk youth participants. Youth participants were aged 14–22 (*M* = 17.7) years and were biologically female.

**Table 1:** Description of Wahine Talk sample (*N* = 35)

**Variable**	***n***	**% of sample**	**M**(***SD***)	**Range**
Age	34		17.8 (*2.6*)	14–22 years
Race and ethnicity				
Native Hawai ‘ian	16	47		
Samoan	8	24		
Tongan	3	9		
Micronesian	10	30		
Other Pacific Islander	4	12		
Chinese	2	6		
Japanese	6	18		
Korean	1	3		
Filipino	7	21		
Other Asian	0	0		
White/Caucasian	3	9		
Hispanic	4	12		
Black/African American	2	6		
Sex				
Female	34	100		
Age at first sex	30	88	14.1 *(1.8)*	12–18 years old
Prior pregnancy	17	50		1–7 pregnancies
Prior miscarriage	11	32		1–6 miscarriages
Prior abortion	6	18		1–2 abortions
Prior live birth	10	30		1–2 births
Children living with participant	8	24		1–2 children
Children living with someone else	2	6		1–2 children
Prior birth control use	6	18		
Birth control use after intervention	20	59		
Foster care				
History of foster care placement	15	44		
Age entered foster care			8.3 *(5)*	1–16 years old

One participant had missing demographic information. Four participants reported not having had sex at baseline

### Data Collection

The study team collected in-depth qualitative data through three focus groups with interdisciplinary teams of Wahine Talk providers, four focus groups with youth participants, and 25 individual interviews with youth participants, for a total of seven Wahine Talk providers and 35 youth participants. The data collection was done in English.

All semi-structured focus group and interview guides asked about linkage to housing services via prompts/probes. The provider focus group guide topics focused on experiences as a provider, evaluation of what did and did not work well, and how to improve Wahine Talk, wherein prompts/probes asked providers about what did/did not work well and could be improved about its linkage to housing services. Participant focus group and interview guide topics focused on experiences being in the program, how to improve Wahine Talk, and the role of one’s culture in attitudes toward sexual health and Wahine Talk. Similarly, prompts/probes asked participants what could be improved about its linkage to housing services. Relative to focus groups, the interviews allowed greater time to ask each participant about wrap-around services such as linkage to housing. The study team transcribed recordings and checked for accuracy prior to analysis.

### Data Analysis

This study employed a type of thematic analysis known as template analysis (Brooks et al., 2015). To begin analysis of the focus group and interview transcripts, the first author developed an initial a priori coding template and its codebook based on reviewing a subset of the transcripts. Then, we conducted coding, revisions to the coding template, and peer debriefing sessions iteratively in multiple rounds. Specifically, the first author coded subsets of the data and then met with the research team for regular peer debriefing sessions. Based on feedback from the debriefing sessions, the initial template was iteratively revised. After all data had been coded in NVivo 10, the first author reviewed the master file of coding results in conjunction with the larger team to produce themes. The full team continued to revise the themes to produce the current manuscript. We conducted comparison analyses across groups (e.g., staff vs. youth) and individuals (e.g., between youth), identifying alternative perspectives. The study enhanced rigor through regular peer debriefing and member checking. To conduct member checking, the first author collected feedback from Wahine Talk staff on two drafts of the manuscript and revised the manuscript accordingly. The first author also met with a Wahine Talk staff to discuss the manuscript data and themes, and revised the analysis and findings accordingly.

### Positionality Statement

The authorship team is built from a research-practice partnership between two universities and a community-based drop-in center for YEH. The team was thus made up of a diverse group of university-based and community-based scholars, including one faculty member (hired as an external evaluator for Wahine Talk’s larger feasibility study), two doctoral students, one undergraduate public health student, and three program providers or administrators from the organization delivering Wahine Talk. Our team was majority female, with diverse racial and ethnic backgrounds (Japanese, Native Hawai ‘ian, other Pacific Islander, Latina, Asian, African American, and White). None of the authors are homeless youth, though all have multiple years (in some cases decades) of experience serving homeless youth in various professional capacities. Four of the seven authors were located in Hawai ‘i (specifically, O ‘ahu) at the time of data collection and analysis. Our roles and identities were continually considered and, at many times, actively leveraged during the process of the study to improve program design and delivery as well as community-engaged data collection and analysis. The authors actively leveraged their roles and identities through reflexive journaling and peer debriefing. During both reflexive journaling and peer debriefing, the authors considered how their lived experiences and identities were influencing their interpretation of the data. For example, a few authors with lived experience in Hawai’i honed in on the data on sweeps in Hawai’i and identified a theme on system-level challenges to linking the youth to housing services.

## Results

The analysis revealed three overall themes on opportunities and challenges to linkage to housing services for youth participants of a comprehensive SRH program for YEH. First, *Building relational trust between staff and youth* discusses having multiple layers of support and staff making one-on-one time for youth. Second*, Instrumental support facilitates linking youth to housing* discusses direct and indirect facilitators. Third, *Local policies and practices challenging linking youth to housing* discusses factors both preventing initiation of linkage to housing services as well as reversing linkage efforts.

### Theme 1: Building Relational Trust Between Staff and Youth is Critical

Wahine Talk youth and staff agreed that building trust between staff and youth was critical to linking youth to housing services. Facilitators to building trust between staff and youth were (i) having multiple layers of support, including housing-specific staff and staff the youth trust, and (ii) staff making one-on-one time for the youth.

#### Having Multiple Layers of Support

Housing-specific staff refers to staff with a specific role providing housing services. In the words of Wahine Talk staff, youth transitioning to stable housing was due to “multiple layers of support,” where one of the layers was having a Housing Placement and Retention Specialist:

There’s two girls who were on LARC, who are now stably housed… Those things come with multiple layers of support and those referrals and getting them connected. Like, now we have, um, an in-house, um, housing…placement and retention specialist. — Staff.

As Wahine Talk staff directly described the positive connection between the youth being stably housed and having the Housing Specialist, it is clear they found the staff beneficial. However, it is not clear if they perceived the benefits as due to the staff having housing-specific expertise or due to having an additional team member to offer time to youth.

The youth did not directly describe their experiences with the Housing Specialist and, while the focus group and interview guided asked about linkage to housing, it did not ask about the Housing Specialist specifically. Thus, it is not possible to assess the youth’s perspective on the Housing Specialist. Potential reasons the youth did not proactively describe their experience with the Housing Specialist is that the youth did not distinguish between the Housing Specialist and other Wahine Talk staff as related to housing — Wahine Talk staff took on multiple roles —, the Specialist was employed for a short duration at Wahine Talk, and/or the Specialist was not a direct Wahine Talk staff person; the Specialist was employed by the overall clinic at YO!.

Both Wahine Talk youth and staff described a positive connection between linkage to housing and having staff who the youth trusted. In the quote below, one of the providers commented on how having staff that the youth trusted “opened up the door…[for] housing”:

… just by them being in the program and they built the trust, it like opened up a lot of doors for other things, like education—and housing. It’s like, “Oh, [staff member], I want this. [Staff member], I want that.” It’s like—it’s just being that person that they go to. It’s just awesome. — Staff.

Youth also referenced specific staff they connected with who helped link youth to housing:

Cuz I was still talking to my worker, and, uh—the one that they referred me to. And she’s like, ‘Okay, well, we’ll help you. We’ll sign you up for the program like to get you housed with Sect. 8.’ …That’s how I’m getting all my benefits, and I have to be working. So with everything from YOs, I would—I would still be on the streets, and they’re the ones that helped me get into the shelter. [Staff name redacted]… She actually vouched for me to go in a shelter…Now I’m in an apartment. — Youth.

Thus, both youth and staff referenced particular staff and youth, respectively, who they had a closer connection with and how the relationship directly facilitated linkage to housing.

While Wahine Talk tracked linkage to housing data, the program curriculum did not specifically ask about the youths’ housing *status* after enrollment. Linkage to housing occurred when staff and/or youth chose to discuss and pursue housing opportunities, as opposed to being a program requirement. The presence of staff youth trusted, thus, was beneficial for youth being linked to housing because it increased the probability of the youth introducing their needs and concerns around housing themselves.

#### Staff Making One-on-One Time for Youth

Although both Wahine Talk youth and staff described having one-on-one time with each other as an opportunity to promote linkage to housing, youth highlighted the need for this more than staff. In particular, youth described the benefits of having informal one-on-one time with the staff. For example, one youth commented:

It would be cool if they could, like, um, like, meet us more, like, individually. ‘Cause I need a lot of help with stuff, like especially my housing, but they only have so many hours that I could be here — Youth.

Thus, the youth cited additional one-on-one time would have provided the space and attention to process housing service-related tasks. The absence of this one-on-one time could reduce the number and quality of conversations around housing for these youth. Although staff did not explicitly state the lack, staff acknowledged the burden on their time and the worry about being able to follow-up sufficiently with youth.

### Theme 2: Instrumental Support Facilitates Linking Youth to Housing

Both the youth and staff also discussed provision of instrumental support as critical for facilitating linking youth to housing. The staff and youth agreed that the program’s provision of drop-in hours with access to basic needs and social services, a checklist, phones for youth participants, and transportation services were key instrumental support factors facilitating linkage to housing, indirectly and directly. While certain factors provided multiple, regular opportunities for meeting and open communication between youth and staff, indirectly leading to conversations on housing, other factors directly helped link youth to housing services.

#### Instrumental Support Indirectly Linking Youth to Housing

A participant described how her journey to being linked to housing through Wahine Talk began with her visiting for basic needs:

Youth: “They helped me with my voucher… housing voucher.”

Interviewer: “Can you talk a little bit about that, how it went?”.

Youth:“Oh, yeah. I never knew about YOs, but then when my brother came along, my sis—um, my brother’s girlfriend, they told me … ‘Oh, yeah, you can get free stuff, and you go to YOs get—go shower, and you can go eat.’ I’m like, ‘What? Are you serious? Are you lying to me? What?’ Free, uh—free diapers. I was like ‘What? You’re lying.’ She was like, ‘No, no, you can.’ I’m like, ‘Nah, I don’t believe you. Well—well, how come I never heard of this place?’”.

In the third year of the program, staff implemented a checklist for the youth to complete. The checklist asked the youth to complete tasks to receive instrumental support (e.g., birth control), and related to peer mentoring, group discussions, and checking in at the center as a prerequisite to receiving an updated phone and/or increased data for the youth’s phone. Below, one of the staff shares how the checklist improved the youth’s discussions with the staff on a range of topics, including housing.

That checklist being a part of the steps to get to them … the nicer the iPhones, right, um, has been really—I think has been a cool way to kind of approach them to keep coming back into YO, um, and to not only connect with them within Wahine Talk, but within education and employment, within housing, within our other basic services. — Staff.

#### Instrumental Support Directly Linking Youth to Housing

The program’s decision to provide cell phones to youth and to allow staff to drive the youth also directly facilitated access to housing services, in addition to fostering communication between youth and staff. A Wahine Talk staff person provided an example of how a youth’s cell phone played a critical part in her being able to discuss and take on a housing subsidy. For youth, they often commented that one of the most beneficial program features was the cell phone, as it improved their ability to communicate. Finally, the Wahine Talk staff said that designing the program to allow some staff to be able to drive the youth also facilitated access to housing services. At times, the staff would drive youth for a housing-specific appointment. Thus, providing cell phones and transportation addressed two barriers to accessing housing services for youth in their community.

### Theme 3: Local Policies and Practices May Challenge Linking Youth to Housing

Wahine Talk staff also discussed broader, system-level factors challenging their ability to connect youth with housing services.

#### Initiation of Linkage to Housing Challenges

A key barrier to not only connecting the youth to housing but to even initiating a conversation about the youth’s housing needs were Hawai ‘i and O ‘ahu’s restricted housing options for unaccompanied minors. Hawai ‘i does not allow unaccompanied minors (i.e., individuals under 18 years without a guardian) into regular shelters. Thus, in many cases, staff did not initiate housing-related conversations with individuals below 18 years given the limited options to pursue housing. A staff explained:

Well, with housing, I think, one of the big challenges that’s—uh, for the kids who are under 18 who are living on their own, there’s not really many options for them. There’s an emergency shelter in the middle of the island, but it’s—kinda the goal is family…family reunification out there, and, um, so that was challenging for the kids under 18. — Staff.

Thus, individuals under 18 years are only allowed in family shelters, of which there is only one on the island of O ‘ahu located 30–45 min from YO!.

#### Reversal of Linkage to Housing Efforts

In addition to the state’s restricted housing options for unaccompanied minors, Honolulu also instates regular police “sweeps” and so-called “compassionate disruption” tactics that reverse the work Wahine Talk youth and staff do to link the youth to housing services. A staff explained:

Since October, they’ve just been sweeping con—constantly, and so it’s like, where we thought they were, they’re not anymore, and it’s on our end to navigate through that and really try to find them. And, um, a lotta times, in the day, they’re hiding cuz they don’t wanna get arrested. — Staff.

While the staff did not explicitly raise the issue of sweeps in connection to linkage to housing, staff stated “a lotta the girls that we just kinda lost connection with and lost” was due to sweeping. Staff also stated:

And so it’s like, where we thought they were, they’re not anymore, and it’s on our end to navigate through that and really try to find them. — Staff.

The sweeps required YEH to clear out from the streets, displacing YEH from their physical locations, and often causing youth to lose their phones. Thus, we interpret that the sweep-induced breaks in communication between the youth and Wahine Talk staff directly challenged staffs’ ability to link youth to housing.

Of note, is that the youth did not comment on the restricted housing options or sweeps as related to housing. It is possible youth did not comment on system-level factors influencing their access to housing services whereas staff did, because system-level influences were easier to identify from the staff’s perspective than the individual youth’s perspective. For example, staff may be able to observe group-level attendance reductions during sweeps while youth are not. Sweeps may also be normalized for youth to the extent that sweeps did not arise as a significant comment during the qualitative data collection. Overall, among youth, those younger than 18 years old were linked to housing less than those 18 or older due to Hawai ‘i’s restricted housing options for unaccompanied minors. In turn, youth younger than 18 years old commented less relative to participants 18 or older on linkage to housing.

## Discussion

The purpose of the current exploratory study was to assess opportunities and challenges to linking young women experiencing homelessness to housing through a comprehensive sexual-reproductive health program (e.g., Wahine Talk). Such comprehensive SRH programs may serve as a critical route to connect YEH to housing outside of the traditional routes of youth entering a homeless shelter or visiting a non-SRH drop-in center, and similar to other non-housing focused settings such as schools. This study makes a unique contribution to the literature, as there is little to no previous research on considerations for linking youth to housing through a comprehensive sexual-reproductive health program, to the authors’ best knowledge. Our exploratory research identified three ways that the opportunities and challenges to linking YEH to housing services could be specific to a comprehensive SRH program as compared to other settings such as a homeless shelter or drop-in only center. Two opportunities could be for the SRH program (1) to have a housing specialist and (2) to provide sufficient opportunities for staff-youth meetings and communication to discuss housing needs. A challenge could be ensuring SRH staff are trained to prioritize youth’s *choice* to reduce or delay pregnancies (i.e. reproductive justice) as opposed to solely prioritizing pregnancy reductions and delays. The findings have implications for future research and interventions aimed at improving access to housing services for youth experiencing homelessness.

### Specific Opportunities to Link YEH with Housing Services Through Comprehensive SRH Programs

Having a housing specialist at an SRH program may provide the same opportunities promoting access to housing from which other non-housing focused organizations benefit. Schools are a community setting which do not have a housing focus, but which have benefited from having a housing-specific staff person to promote access to housing services for their youth. Similarly, a housing-specific staff at a comprehensive SRH program can be an opportunity to promote access to housing even if the individual is not exclusively focused on housing access issues. Further study on whether having a housing specialist increases youth visits or positive health outcomes and, if so, through what mechanisms is warranted. For example, if the housing specialist leads to improved implementation or evaluation outcomes, it is beneficial to understand if the benefits are due to the staff’s housing expertise or for alleviating the program’s overall caseload.

Due to comprehensive SRH programs not having a primary focus on housing, there is a specific need for SRH programs to provide multiple opportunities for youth and staff to meet and communicate, making housing-focused conversations more probable. As discussed by staff, these conversations are facilitated by sufficient provision of drop-in hours for basic needs, a checklist, phones, and pick-up rides for youth participants. Participants both stated the drop-in hours was one of the features they liked and had no issues with, while others recommended providing more drop-in hours. Given participants’ feedback and given this SRH program offered an average of 12 h per week (as noted in the Introduction), a possible recommendation is for similar SRH programs to offer a minimum of 12 h of drop-in a week.

There is also a distinction between efforts which promote youth *ever* visiting the SRH program, and efforts to improve the chances of the youth returning to the center and developing a connection with the staff, wherein they share their housing needs with staff. Previous research indicates that youth are more likely to ever visit a drop-in center if the center provides hygiene-related needs such as showers, food, and clean clothes (Tucker et al., 2018). However, of youth who ever visited the drop-in center, their attendance at the center could be more frequent when the youth report staff as individuals they can trust and count on for support (Tucker et al., 2018). Our exploratory study findings suggest varied opportunities for communication such as instating peer mentoring requirements and providing youth with a cell phone to promote linkage to housing. Further study on which program components were associated with greater linkage to housing and by how much would be beneficial.

### Specific Challenges Linking YEH with Housing Services Through Comprehensive SRH Programs

Finally, staff stated it was challenging to house YEH given Oahu has one shelter for unaccompanied minor YEH. In the context of comprehensive SRH programs, the challenge of housing YEH given the limited housing options may be particularly exacerbated because SRH programs typically discourage new pregnancies (e.g., for non-parenting youth) and encourage reduced or delayed pregnancies, meaning SRH program youth participants are less likely to qualify for the housing options designed for families (e.g. parenting youth).

Prior research on reproductive justice is tied to the challenge of linking YEH to housing services given the limited housing options. In a separate analysis utilizing PhotoVoice data, Wahine Talk youth participants reported that prioritizing reproductive justice, which refers to protecting the youth’s *choice* to reduce, delay, or have pregnancies, is a critical part of a comprehensive SRH program and that Wahine Talk and Wahine Talk staff prioritized the youths’ reproductive justice (Aparicio et al., 2021). Prior research also demonstrates that women experiencing homelessness who gave birth as an adolescent commonly experience trauma due to not being able to be part of the decision-making process around their reproductive health and having their patient rights violated through coercion (Cronley et al., 2018). Thus, an implication of the current findings on challenges linking YEH to housing services is that, at the program-level, SRH programs’ mission should be to ensure reproductive justice instead of pregnancy reduction. Second, at the program and staff-level, programs should ensure SRH staff are trained to protect youth’s reproductive choice in lieu of prioritizing pregnancy reductions/delays alone.

Previous research finds that being pregnant can be a transformative event in a young individual’s life and a time when youth seeking housing services (Smid et al., 2010). Thus, in line with prioritizing youths’ reproductive choice, SRH program missions should incorporate flexibility to view the youth’s pregnancy as the youth’s strength instead of a limitation in the context of accessing housing services.

### Limitations

Findings from this study should be interpreted within the context of its limitations. The primary limitation is that the data collection instruments only had a secondary aim of examining the SRH programs’ access to housing and other wraparound services. Thus, while all the data collection instruments contained probes on linkage to housing, they did not contain relevant questions such as those on the process of being linked to housing. Second, it is important to note the generalizability, or transferability, of our findings. The findings on opportunities and challenges to link YEH to housing are transferrable to other programs or community settings designed similar to Wahine Talk. For example, if a comprehensive SRH program does not co-locate its services like Wahine Talk, facilitators, and challenges to providing housing services may be distinct. Similarly, the current findings are transferrable to a youth homelessness environment politically, culturally, and socioeconomically similar to O ‘ahu. For example, O ‘ahu only has one youth homeless shelter and Honolulu has regular police “sweeps” and “compassionate disruption” tactics, which displace youth experiencing homelessness. In areas of the country with more youth shelters or less sweeps, different levels and types of system-level challenges linking youth to housing are expected. Areas with less sweeps are likely to have less system-level challenges because communication between SRH staff and youth is expected to be more consistent. As sweeps have been shown to drain city budgets and further displace those experiencing homelessness without addressing the root causes of homelessness, areas with less sweeps are expected to have better budgets to address the root causes of homelessness. These considerations should be evaluated when applying the findings to other YEH and/or program contexts.

## Conclusion and Implications

Our findings suggest that comprehensive SRH programs may have opportunities and challenges to linking YEH to housing which are consistent with other programs such as shelters and non-SRH drop-in centers. However, SRH programs also have specific opportunities and challenges to ensuring their youth participants can access housing services. In particular, SRH programs may benefit from employing housing-specific staff and from providing sufficient opportunities for staff-youth meetings and communication. Additionally, SRH programs may have a specific challenge in ensuring staff are sufficiently trained to prioritize youth participants’ reproductive choice in lieu of pregnancy reductions/delays alone.

## Data Availability

All coding templates (i.e., for Nvivo coding) generated during the current study are not publicly available but are available from the corresponding author on reasonable request. The software applications used support the published claims.

## Data Availability

De-identified data may be available on reasonable request, in conjunction with an executed data use agreement.
