# Correction: Opportunities and Challenges to Linkage to Housing in the Context of a Sexual and Reproductive Health Program for Youth Experiencing Homelessness

**DOI:** 10.1007/s11121-023-01629-8

**Published:** 2023-12-22

**Authors:** Maisha R. Huq, Danielle R. Phillips, Christine Childers, Rebecca Chavez, Jacqueline Tellei, Lenora Blakely, Elizabeth M. Aparicio

**Affiliations:** 1 Department of Behavioral and Community Health, School of Public Health, University of Maryland, College Park, 4200 Valley Drive, 20742 College Park, MD USA; 2 School of Social Work, University of Maryland, Baltimore, MD USA; 3 Waikiki Health, DOD Department of Public Health, Fayetteville, NC USA; 4 Waikiki Health, Honolulu, HI USA; 5 PATH Clinic, Waikiki Health, Honolulu, HI USA; 6 Community THRIVES Lab, Department of Behavioral and Community Health, School of Public Health, University of Maryland, College Park, MD USA

**Correction to: Prevention Science** 10.1007/s11121-023-01560-y

The article “Opportunities and Challenges to Linkage to Housing in the Context of a Sexual and Reproductive Health Program for Youth Experiencing Homelessness”, written by Huq, M.R., Phillips, D.R., Childers, C., Chavez, R., Tellei, J., Blakely, L., and Aparicio, E.M., was originally published electronically on the publisher’s internet portal on 10 June 2023 without open access. With the author(s)’ decision to opt for Open Choice the copyright of the article changed on 06 December 2023 to © The Author(s) 2023 and the article is forthwith distributed under a Creative Commons Attribution 4.0 International License, which permits use, sharing, adaptation, distribution and reproduction in any medium or format, as long as you give appropriate credit to the original author(s) and the source, provide a link to the Creative Commons licence, and indicate if changes were made. The images or other third party material in this article are included in the article’s Creative Commons licence, unless indicated otherwise in a credit line to the material. If material is not included in the article’s Creative Commons licence and your intended use is not permitted by statutory regulation or exceeds the permitted use, you will need to obtain permission directly from the copyright holder. To view a copy of this licence, visit http://creativecommons.org/licenses/by/4.0/.

The original article has been corrected.

